# A review of the use of covariates in cluster randomized trials uncovers marked discrepancies between guidance and practice

**DOI:** 10.1016/j.jclinepi.2014.12.006

**Published:** 2015-06

**Authors:** Neil Wright, Noah Ivers, Sandra Eldridge, Monica Taljaard, Stephen Bremner

**Affiliations:** a Centre for Primary Care and Public Health, Blizard Institute, Queen Mary University of London, Yvonne Carter Building, 58 Turner Street, London, E1 2AB, United Kingdom; b Family Practice Health Centre and Institute for Health Systems Solutions and Virtual Care, Women's College Hospital, 76 Grenville Street, Toronto, ON M5S1B2, Canada; c Department of Family and Community Medicine, University of Toronto, 500 University Avenue, 5th Floor, Toronto, ON M5G1V7, Canada; d Clinical Epidemiology Program, Ottawa Hospital Research Institute, Ottawa Hospital, Civic Campus, 1053 Carling Avenue, Civic Box 693, Ottawa, Ontario K1Y 4E9, Canada; e Department of Epidemiology and Community Medicine, University of Ottawa, Ottawa, Ontario, Canada

**Keywords:** Cluster randomized trials, Covariates, Restricted randomization, CONSORT statement, Adjusted analyses, Baseline imbalance

## Abstract

**Objectives:**

Reviews of the handling of covariates in trials have explicitly excluded cluster randomized trials (CRTs). In this study, we review the use of covariates in randomization, the reporting of covariates, and adjusted analyses in CRTs.

**Study Design and Setting:**

We reviewed a random sample of 300 CRTs published between 2000 and 2008 across 150 English language journals.

**Results:**

Fifty-eight percent of trials used covariates in randomization. Only 69 (23%) included tables of cluster- and individual-level covariates. Fifty-eight percent reported significance tests of baseline balance. Of 207 trials that reported baseline measures of the primary outcome, 155 (75%) subsequently adjusted for these in analyses. Of 174 trials that used covariates in randomization, 30 (17%) included an analysis adjusting for all those covariates. Of 219 trial reports that included an adjusted analysis of the primary outcome, only 71 (32%) reported that covariates were chosen a priori.

**Conclusion:**

There are some marked discrepancies between practice and guidance on the use of covariates in the design, analysis, and reporting of CRTs. It is essential that researchers follow guidelines on the use and reporting of covariates in CRTs, promoting the validity of trial conclusions and quality of trial reports.

**What is new?**

**Key findings**
• Restricted randomization was used in around half of cluster randomized trials (CRTs).
• Few trial reports included tables summarizing both cluster-level and individual-level baseline covariates, and over half of trials reported a significance test of baseline balance.
• Adjusted analyses were common in CRTs, but authors often did not report whether covariates have been chosen before or after seeing the data.

**What this adds to what was known?**
• There are some marked discrepancies between practice and guidance in the use of covariates in CRTs.

**What is the implication and what should change now?**
• Researchers and editors should note existing guidelines on the use and reporting of covariates in CRTs.

## 1 Introduction

In cluster randomized trials (CRTs), preexisting groups (clusters) of individuals (eg, family practices, hospitals, communities, or schools) are randomized to intervention or control treatment arms. This is in contrast to individually randomized trials, where independent individuals are randomly allocated to treatment arms. A cluster randomized design may be used for one or more of several reasons, including logistical or administrative convenience; to avoid contamination; to improve compliance with treatment; to enable the use of routine data only available at the cluster level; or to evaluate an intervention that is applied at the cluster level. Covariates are commonly used in the design and analysis of randomized trials to promote balance between treatment arms and to improve precision and power [1,2]. Unlike in individually randomized trials, covariates in CRTs can exist at two levels that of the cluster and that of the individual. A cluster-level covariate describes an intrinsic characteristic of the cluster and is fixed for all individuals in the same cluster. An individual-level covariate describes something about an individual and may vary across individuals in the same cluster.

Specific recommendations are available with respect to the use of covariates in the design and analysis of randomized controlled trials. For example, the CONSORT guidelines [3] include recommendations for the reporting of covariates, and there is published guidance on adjusting for covariates in analyses [1,4]. Austin et al. [5], Assmann et al. [6], and Yu et al. [7] have reviewed adherence to recommendations with respect to the handling of covariates in randomized trials, but reviews have excluded CRTs. A review of CRTs in primary care [8] included the use of matching or stratification and reporting of baseline covariates. In this review, we extend the work of Austin et al. [5] to CRTs, where the multilevel nature of CRTs introduces additional complexity in the handling of covariates.

CRTs usually randomize a smaller number of units, so there is a greater risk of imbalance of covariates between treatment arms. The use of restricted randomization, via stratification, matching, or minimization, is therefore recommended to help balance both cluster-level covariates and individual-level covariates summarized at the cluster level, when available, between treatment arms [9]. Failure to do so may be a missed opportunity to improve validity and power by promoting balance in prognostic covariates [2,9].

The CONSORT extension for CRTs [10] recommends reporting both cluster-level and individual-level covariates as applicable in tables comparing baseline characteristics between arms. Failure to report baseline distributions of covariates limits the extent to which a reader of a trial report can assess the validity of a trial's findings and external relevance. There are clear recommendations against significance testing of balance of covariates between treatment arms [1,11].

Including covariates that are strongly correlated with outcome in an analysis can improve analytic power and statistical precision [1,12,13]. A baseline measure of the outcome variable is often strongly correlated with outcome and should be included in an adjusted analysis because it represents an opportunity to improve the statistical power of the analysis [14]. Covariates used in randomization should also be adjusted for in an analysis [1,4].

The effects of adjusting for covariates in CRTs are more complex than in individually randomized trials, as adjusting for covariates may affect both the cluster-level and individual-level residual variance of outcome. Only limited guidance for choosing covariates in the analysis of CRTs has been published [2,14]. As for individually randomized trials, choosing covariates post hoc can invalidate conclusions, although failure to report the method and justification of choosing covariates prevents readers from assessing the validity of adjusted analyses [4,11].

In this article, we review a random sample of published CRTs with respect to the use of covariates in randomization, the reporting of covariates by treatment arm, and adjustment for covariates in analyses of the primary outcome. In particular, we assess whether: (1) covariates were used in randomization, (2) tables of baseline cluster-level and individual-level covariates were presented, (3) significance tests for baseline balance of covariates were avoided, (4) an adjusted analysis of the primary outcome was reported, (5) an analysis adjusting for baseline measure of the outcome (if available) was reported, (6) an analysis adjusting for all covariates used in randomization was reported, and (7) all covariates were reported to have been chosen a priori.

## 2 Methods

### 2.1 Trial sample

This review used a random sample of 300 CRTs previously identified using a published electronic search strategy [15] implemented in MEDLINE. This sample has been used to assess the impact of the CONSORT extension [10] on the reporting and methodological quality of CRTs [16]. The sample includes CRTs for which the main report was published between 2000 and 2008, across 150 English language journals. The journals include general medical journals [eg, the New England Journal of Medicine (NEJM), the Journal of the American Medical Association (JAMA), The Lancet, and the British Medical Journal (BMJ)] and various specialty journals (eg, the British Journal of Psychiatry, Diabetes Care, the International Journal of Cancer, and the Journal of Nutrition).

### 2.2 Data abstraction

Two reviewers (N.W. and N.I.) independently read and abstracted a defined list of items from each article. Any discrepancies were resolved by consensus. The review method was piloted on 15 articles, leading to clarifications to the abstraction strategy. Twenty-five items were extracted from each trial report, relating to: sample size and primary outcome type (three items), use of covariates in randomization (four items), reporting covariates at baseline (six items), adjusting for covariates in an analysis of the primary outcome (eight items), and selection of covariates (two items). Ten items related directly to recommendations made for handling covariates; others were abstracted to provide general background characteristics of the trial and information about the use of covariates. Definitions of variables abstracted and justification are described in the following paragraphs.

### 2.3 Data abstracted

We describe each covariate as either a cluster- or individual-level variable. The level is that at which the covariate is most naturally measured. This may not always be the level at which it is reported or used in analysis. For example, in a trial where general practitioners are the clusters and their patients are the individuals, the number of years since registration as a general practitioner is a cluster-level covariate, whereas the age of a patient is an individual-level covariate. A covariate measured entirely or mostly on individuals not taking part in the trial and used to describe some aspect of the cluster would be counted as a cluster-level covariate. For example, in a trial where the cluster is a general practice and only some patients in the practice are recruited to the trial, the average age of all patients in a general practice is a cluster-level covariate. If a single covariate was summarized and reported in multiple ways (eg, mean age and proportion of patients above age 65), it was counted as one covariate.

We limited abstraction to analyses of the primary outcome for each trial. The primary outcome was defined to be the first primary outcome identified by the author. If a primary outcome was not identified by the author, then it was identified as: the outcome used in the sample size calculation, the outcome identified by the article's title, or the first reported outcome in the article. To be included in this review, an analysis had to include an estimate of treatment effect plus a measure of uncertainty (such as a confidence interval or standard error) or a *P*-value of a significance test of no treatment effect.

We characterized covariates used in analyses as one of the following: baseline measure of primary outcome, covariate used in randomization (eg, stratification, minimization, or matching factors), and any other covariate. When a study reported change from baseline as the primary outcome, we counted this as an attempt to adjust for the baseline measure of outcome, acknowledging that this is not the recommended approach [1]. We considered covariates used in randomization separately from other covariates because these should be adjusted for in the analysis [1,4] and to enable comparison with previous methodological reviews [5].

When considering analyses adjusted for covariates other than the baseline measure of outcome or covariates used in randomization, only the analysis identified by the author as the main adjusted analysis (or the first reported if a main adjusted analysis was not identified) was evaluated. Subgroup analyses were not considered. Any reason given for selecting covariates for multivariate analysis was considered as justification given.

The reported method for selecting covariates for adjusted analyses was considered post hoc if the article described a method where covariates were chosen after observation of any trial data. For example, selecting covariates due to an observed imbalance between treatment groups, or using a covariate selection algorithm, were post hoc methods. If the article reported that covariates were chosen before any data were observed, for example, they were prespecified in an analysis plan, then the reported method of choosing covariates is a priori. In all other cases, the method was considered unclear.

## 3 Analysis

Results are presented using descriptive statistics and include two sets of results by subgroups, which were chosen post hoc for further investigation. We explored whether the use of covariates in randomization and adjusting for covariates at cluster and individual level varied according to the number of clusters in the trial. We explored whether reporting covariates in baseline tables, using statistical tests of baseline balance, and reporting choosing covariates a priori differed for trials published in high-impact journals compared with other journals.

In the first stratified analysis, three subgroups were defined by mean number of clusters per treatment arm. Subgroups were formed by the lower quartile, upper quartile, and remaining trials: five or fewer clusters per arm, from 5.5 to 23 clusters per arm, and more than 23 clusters per arm. These subgroups contained 75, 147, and 71 trials, respectively. In this analysis, seven trials were excluded as the mean number of clusters per treatment arm could not be calculated because either the total number of individuals or the number of clusters in the trial was not reported.

In the second stratified analysis, two subgroups were defined by the impact factor of the publishing journal. Impact factors were obtained from journal citation reports (ISI Web of Science, 2009). We defined high-impact journals as those with impact factor greater than 10, which in this sample are the New England Journal of Medicine (NEJM); the Journal of the American Medical Association (JAMA); The Lancet; Annals of Internal Medicine; PLOS Medicine; and the British Medical Journal (BMJ). Fifty-one trial reports in the sample were published in these high-impact journals. The remaining 249 trial reports were published in other journals.

## 4 Results

### 4.1 Trial characteristics

Characteristics of the 300 trial reports used in this review are summarized in table number 2 of Ivers et al. [16].

### 4.2 Randomization

Details with respect to the use of covariates in randomization are presented in Table 1. In 174 (58.0%) of the 300 trials, at least one covariate was used in randomization. In 130 trials (43.3%), at least one covariate other than cluster location and cluster size was used. In 118 trials (39.3%), only cluster-level covariates were used.

In the 124 trials that used cluster-level covariates in randomization, 74 (59.7%) used one cluster-level covariate, 24 (19.4%) used two, and 26 (21.0%) used between three and nine. Only 12 trials used individual-level covariates in randomization of which nine (75%) used only one individual-level covariate.

Of 277 trials that were conducted in more than one location, authors of 50 (18.1%) attempted to balance for cluster location in randomization (3 of 277 were unclear). Of 285 trials with varying cluster sizes, authors of 51 (17.9%) attempted to balance cluster size in randomization (4 of 285 were unclear). Of 263 trials with multiple location and variable cluster size, authors of 12 (4.6%) attempted to balance both location and cluster size in randomization.

### 4.3 Reporting of baseline covariates

Of 300 trial reports, 69 (23.0%) included tables reporting both cluster- and individual-level covariates by treatment arm, whereas 158 (52.7%) included a table of individual-level covariates only. Further details are given in Table 1. The number of cluster-level covariates described in a table or in the text ranged from 1 to 19 (if nonzero), with a median of 3. The number of individual-level covariates described in a table or in the text ranged from 1 to 28 (if nonzero), with a median of 7.

Of 300 trial reports, 207 (69%) reported a baseline measure of the primary outcome. A significance test of the balance of a covariate between treatment arms was reported or referred to in 166 trial (55.3%) reports.

### 4.4 Adjusted analyses

Of 300 trial reports, 219 (73.0%) included at least one adjusted analysis of the primary outcome. Of 207 trial reports that reported a baseline measure of the primary outcome, 155 (74.9%) included an analysis adjusting for a baseline measure of the outcome. Of 174 trials that used covariates (including cluster size and location) in randomization, 30 (17.2%) included an analysis adjusting for all covariates used in randomization. These results are summarized in Table 1. Seven trial reports did not include any unadjusted or adjusted analysis of the primary outcome, according to our criteria.

There were 140 trials (46.7%) that included an analysis adjusting for covariates other than baseline measure of outcome or covariates used in randomization. Of these 140 adjusted analyses, 7 (5.0%) included only cluster-level covariates, whereas 100 (71.4%) adjusted for only individual-level covariates. In 29 analyses (20.7%), both cluster- and individual-level covariates were included. In four (2.9%) of these trials, it was unclear which level of covariates had been included. When included, the number of cluster-level covariates ranged from 1 to 8, with a median of 1. Likewise, the number of individual-level covariates included in an adjusted analysis ranged from 1 to 28, with a median of 3.

### 4.5 Choosing covariates

Of the 219 trial (73.0%) reports that included an adjusted analysis of the primary outcome, in 71 authors (32.4%) reported choosing covariates a priori, but in 93 (42.4%), it was not reported when covariates adjusted for had been chosen. In 73 trial (33.3%) reports, authors gave some justification for the choice of covariates.

### 4.6 Subgroup results—number of clusters

The results from the subgroup analyses based on trial size are summarized in Table 2. At least one covariate other than cluster location and cluster size was used in randomization in 22 of 75 trials (29%) with five or fewer clusters per treatment arm. In trials with more than 23 clusters per treatment arm, 28 of 71 (39%) balanced on covariates other than cluster location or cluster size. In the remaining 147 trials, 79 (53.7%) used other covariates in randomization.

Thirty-three (44%) of the 75 smallest trials included an analysis adjusting for covariates other than baseline measure of outcome and covariates used in randomization. Five (15%) of those adjusted for cluster-level covariates. Of the 71 largest trials (more than 23 clusters per arm), 34 (48%) reported an analysis adjusting for other covariates, and 10 (29%) of those analyses used cluster-level covariates. Of the 147 remaining trials, 71 (48%) included an analysis adjusting for other covariates, with 21 (30%) using cluster-level covariates.

### 4.7 Subgroup results—journal impact factor

The results described in this section are summarized in Table 3. Reports published in journals with high-impact factors showed higher adherence to three guidelines assessed. In particular, tables reporting both cluster- and individual-level covariates were included in 24 of 50 trial reports (48%) in high-impact journals, compared with 45 of 250 trial reports (18.0%) in other journals; a significance test of balance of a covariate between treatment arms was reported or referred to in 17 of 50 trial reports (34%) in high-impact journals, compared with 149 of 250 trial reports (59.6%) in other journals; and 7 (21%) included an analysis adjusting for all covariates used in randomization compared with 23 (16.3%) in other journals.

Of 30 trial reports in high-impact journals that reported a baseline measure of the primary outcome, 21 (70%) included an analysis adjusting for a baseline measure of outcome, compared with 134 (75.7%) of 177 in other journals. In 19 high-impact factor journal reports (51.4%), it was unclear when covariates adjusted for in the analysis had been chosen compared with 74 (40.7%) in other journals.

## 5 Discussion

In CRTs, baseline balance of covariates may be a greater concern than in individually randomized trials [9]. Despite recommendations to use restricted randomization, especially in smaller CRTs [9], over 40% of authors did not do so. This compares to 64% of authors not using restricted randomization in a review of CRTs in primary care [8]. Furthermore, attempting to use restricted randomization to achieve balance on individual-level covariates was rare, although balance of both cluster-level and individual-level covariates is important in a CRT [9].

Although 80% of trials included at least one table reporting covariates by treatment group, less than 25% included tables for both cluster- and individual-level covariates at baseline, as recommended by CONSORT guidelines [10]. The proportion of trial reports including a table of individual-level covariates by treatment arm at baseline was slightly higher in high-impact journals (84%). This compares to 96.5% and 96% in the individually randomized trials reviewed by Austin et al. [5] and Saquib et al. [17], respectively. Those reviews included only articles published in selections of journals with high-impact factors. This suggests greater adherence in trial reports in high-impact journals, rather than a difference in reporting between CRTs and individually randomized trials.

More than half of sampled CRTs (55.3%) reported a significance test of baseline balance, despite clear recommendations against this practice [1,11]. Although these recommendations strictly apply to individually randomized trials, the argument extends directly to not testing baseline balance of covariates that can only be imbalanced by chance. This practice appears less common in high-impact journal reports (34%) and compares to 38.2% and 46% in the reviews of individually randomized trials by Austin et al. [5] and Saquib et al. [17], respectively.

At least one adjusted analysis of the primary outcome was reported in 73% of CRT reports. Adjusted analyses of the primary outcome using the baseline measure of outcome (as recommended by Murray [14]) were conducted in 74.9% of trials that reported baseline values of the outcome. An analysis adjusting for other covariates (not baseline measure of outcome or covariates used in randomization) was reported in almost half (46.7%) of trial reports. This compares to 34.2% of individually randomized trials in the review by Austin et al. [5], which included analyses adjusting for covariates other than baseline measure of outcome and covariates used in randomization.

In one-quarter of adjusted analyses, some or all covariates were reportedly chosen post hoc, defying guidance for choosing all covariates a priori [2]. In 93 cases (42.5%), it was not reported when the covariates adjusted for in the analysis had been chosen. This does not allow any assessment of the validity of selecting covariates but is less common than in the individually randomized trials considered by Austin et al. [5] and Yu et al. [7] (67% and 75.6% of trial reports). Only a third of CRT reports gave any justification for covariate choice, compared with 41.7% and 73.6% of individually randomized trials in the reviews by Assmann et al. [6] and Yu et al. [7], respectively.

This review is limited by the age of the included trial reports, ranging from 6 to 14 years old. It is plausible that more recently published CRTs could show different characteristics. In addition, we limited abstraction to one primary outcome and one adjusted analysis from each trial report. Therefore, the number of adjusted analyses actually conducted is almost certainly greater than we report. We did not carry out any hypothesis testing or inferential analysis, as we sought to describe practice and had no a priori hypotheses. Similarly, our results by subgroup were descriptive as we had no a priori hypotheses. Finally, no inferences were made with respect to the appropriateness of methods used for randomization or adjusting for covariates in any trial as there is uncertainty regarding ideal strategies.

In summary, there are some marked discrepancies between practice and guidance for CRTs with regard to using covariates in randomization, reporting covariates, testing balance, and methods of choosing covariates. As with the reviews of individually randomized trials, there is inadequate adherence to clear guidance such as not using statistical tests of baseline balance of covariates and choosing covariates to be used in an adjusted analysis a priori. There appears to be better adherence to some recommendations among CRTs published in high-impact journals, compared with CRTs reported in other journals. Recommendations to use restricted randomization and to report both cluster-level and individual-level covariates are not well followed. It is essential that researchers conducting CRTs follow existing guidelines on the use and reporting of covariates to ensure validity of trial conclusions and aid readers in assessing the quality and results of a trial. Readers should be aware of limitations in trials that do not adhere to such guidelines. Further research is needed into the effects of adjusting for cluster- and individual-level covariates in the analysis of CRTs, and further guidance is needed for choosing covariates in CRTs.

## Figures and Tables

**Table 1:** Use of covariates in randomization, reporting of covariates, and use of covariates in analysis

Review item	Number of trials/relevant trials (%)	Note
Use of covariates in randomization		
Covariates used in randomization	174/300 (58.0)	(3/300 unclear)
Types of covariates used in randomization		
Cluster location	50/277 (18.1)	(3/277 unclear)
Cluster size	51/285 (17.9)	(4/285 unclear)
Other		
Only cluster-level covariates	118/300 (39.3)	
Only individual-level covariates	6/300 (2.0)	
Both cluster- and individual-level covariates	6/300 (2.0)	
None	167/300 (55.7)	
Unclear	3/300 (1.0)	
Reporting of covariates		
Trial report includes a table reporting		
Cluster-level covariates only	13/300 (4.3)	
Individual-level covariates only	158/300 (52.7)	
Cluster- and individual-level covariates	69/300 (23.0)	
None	60/300 (20.0)	
Baseline measure of primary outcome reported	207/300 (69.0)	
Significance test of balance of a covariate between treatment arms	166/300 (55.3)	
Covariates used in analysis		
Trial report includes		
An unadjusted analysis of the primary outcome	130/300 (43.3)	
At least one adjusted analysis of the primary outcome	219/300 (73.0)	
Trial report includes an analysis of the primary outcome adjusting for		
Baseline measure of outcome	155/207 (74.9)	
All covariates used in randomization	30/174 (17.2)	
Other covariates	140/300 (46.7)	
When covariates were reported to be chosen		
A priori	71/219 (32.4)	
Post hoc	37/219 (16.9)	
Both a priori and post hoc	18/219 (8.2)	
Not reported or unclear	93/219 (42.5)	
Justification given for the selection of covariates	73/219 (33.3)	

**Table 2:** Covariates used in randomization and covariates used in an adjusted analysis, by subgroup of mean number of clusters per treatment arm

Review item	Mean number of clusters per treatment arm		
	1 to 5 (%)	5.5 to 23 (%)	23.5 to 3,510.5 (%)
Types of covariates used in randomization (excluding cluster location and cluster size)			
Only cluster level	18/75 (24.0)	73/147 (49.7)	26/71 (36.6)
Only individual level	2/75 (2.7)	3/147 (2.0)	1/71 (1.4)
Both cluster and individual level	2/75 (2.7)	3/147 (2.0)	1/71 (1.4)
None	52/75 (69.3)	66/147 (44.9)	43/71 (60.6)
Unclear	1/75 (1.3)	2/147 (1.4)	0/71 (0.0)
Covariates (other than baseline measure of outcome and covariates used in randomization) included in an adjusted analysis			
Cluster only	0/33 (0.0)	1/71 (1.4)	6/34 (17.6)
Individual only	25/33 (75.8)	50/71 (70.4)	23/34 (67.6)
Both cluster and individual level	5/33 (15.2)	20/71 (28.2)	4/34 (11.8)
Unclear	3/33 (9.1)	0/71 (0.0)	1/34 (2.9)

**Table 3:** Reporting of covariates and use of covariates in analysis, by subgroup defined by journal impact factor

Review item	High-impact journals (impact factor >10) (%)	Other journals (%)
Reporting of covariates		
Trial report includes a table reporting		
Cluster-level covariates only	2/50 (4.0)	11/250 (4.4)
Individual-level covariates only	18/50 (36.0)	140/250 (56.0)
Cluster- and individual-level covariates	24/50 (48.0)	45/250 (18.0)
None	6/50 (12.0)	54/250 (21.6)
Significance test of balance of a covariate	17/51 (34.0)	149/250 (59.6)
Covariates used in analysis		
Trial report includes an adjusted analysis of the primary outcome	37/50 (74.0)	182/250 (72.8)
Trial report includes an analysis of the primary outcome adjusting for		
Baseline measure of outcome	21/30 (70.0)	134/177 (75.7)
All covariate used in randomization	7/33 (21.2)	23/141 (16.3)
Other covariates	22/50 (44.0)	118/250 (47.2)
When covariates were reported to be chosen		
A priori	12/37 (32.4)	59/182 (32.4)
Post hoc	5/37 (13.51)	32/182 (17.6)
Both a priori and post hoc	1/37 (2.7)	17/182 (9.3)
Unclear	19/37 (51.4)	74/182 (40.7)
Justification given for the selection of covariates	12/37 (32.4)	61/182 (33.5)
